# Differential effects, on oncogenic pathway signalling, by derivatives of the HNF4 α inhibitor BI6015

**DOI:** 10.1038/s41416-018-0374-5

**Published:** 2019-02-22

**Authors:** Jin-Hee Kim, Hyo Jin Eom, GyuTae Lim, Sungjin Park, Jinhyuk Lee, Seungyoon Nam, Yon Hui Kim, Jin-Hyun Jeong

**Affiliations:** 1 College of Pharmacy, Yonsei Institute of Pharmaceutical Sciences, Yonsei University, Incheon, 21983 Korea; 2 Research and Development Department, Corestem Inc., 24 Pangyo-ro, 255beon-gil, Bundang-gu, Seongnam-si, Gyeonggi-do, 13486 Korea; 3 Genome Editing Research Center, Korea Research Institute of Bioscience and Biotechnology (KRIBB), Daejeon, 34141 Korea; 4 Department of Bioinformatics, KRIBB School of Bioscience, Korea University of Science and Technology (UST), Daejeon, 34113 Korea; 5 Gachon Institute of Genome Medicine and Science, Gachon University Gil Medical Center, Incheon, 21565 Korea; 6 College of Medicine, Gachon University, Incheon, 21565 Korea; 7 Department of Life Sciences, Gachon University, Seongnam, 13120 Korea; 8 Department of Biomedical Science, Hanyang Biomedical Research Institute, Hanyang University, Seoul, 04763 Korea

**Keywords:** Networks and systems biology, Chemical modification

## Abstract

**Background:**

Gastric cancer (GC) is a highly heterogeneous disease with few “targeted” therapeutic options. Previously, we demonstrated involvement of the transcription factor HNF4α in human GC tumours, and the developmental signal mediator, WNT5A, as a prognostic GC biomarker. One previously developed HNF4α antagonist, BI6015, while not advancing beyond preclinical stages, remains useful for studying GC.

**Methods:**

Here, we characterised the antineoplastic signalling activity of derivatives of BI6015, including transfer of the nitro group from the *para* position, relative to a methyl group on its benzene ring, to the *ortho*- and *meta* positions. We assessed binding efficacy, through surface plasmon resonance and docking studies, while biologic activity was assessed by antimitogenic efficacy against a panel of GC cell lines, and dysregulated transcriptomes, followed by pathway and subpathway analysis.

**Results:**

The *para* derivative of BI6105 was found substantially more growth inhibitory, and effective, in downregulating numerous oncogenic signal pathways, including the embryonic cascade WNT. The *ortho* and *meta* derivatives, however, failed to downregulate WNT or other embryonic signalling pathways, unable to suppress GC growth.

**Conclusion:**

Straightforward strategies, employing bioinformatics analyses, to facilitate the effective design and development of “druggable” transcription factor inhibitors, are useful for targeting specific oncogenic signalling pathways, in GC and other cancers.

## Background

Gastric cancer (GC) is the fifth-most prevalent cancer in the world, and the fourth-leading cause of cancer death.^1^ Like most cancers, early detection is paramount, offering the most promising outcomes, via surgical debulking, to minimise residual disease. GC incidence and mortality rates are, by far, the highest in East Asia, largely for unknown reasons.^2^ However, even while GC is relatively rare in most Western countries, population screening is not routine, resulting in delayed diagnosis and poor prognosis (e.g., 5-year survival of merely 30% in the United States, and 19% in the United Kingdom).^3^ Consequently, more effective therapies are urgently needed. As GC is a highly heterogeneous malignancy,^4^ personalised medicine (i.e., stratification of patients most likely to respond to specific therapies) is currently the best option to prolong survival. To date, however, most GC clinical trials, of various targeted therapies, have been disappointing.^5–7^

It is now largely accepted that most solid tumours derive from a subpopulation of stem-like cells reliant on embryonic signalling cascades, including the well-known pathway WNT.^8^ Moreover, it has been hypothesised that WNT signalling is activated by the bacterium *Helicobacter pylori*, a major risk factor for GC.^9^ Clinical studies of WNT inhibitors, however, have thus far been quite limited, and demonstrated only modest antitumour activity,^10,11^ and one possible interpretation is that its downstream effectors (e.g., TCF/LEF, HNF4α) are impinged upon by “cross-talk” from other mitogenic pathways.^12,13^ Thus, while still in its infancy, an increasing strategy is the development of potential antagonists of oncogenic transcription factors.^14–16^

Previously, we demonstrated that a specific subset of GCs (primarily diffuse type) upregulate their metabolism, via an AMPK-HNF4α-Wnt5A transduction cascade, with an HNF4α antagonist, BI6015 being antitumorigenic, both in vitro and in vivo.^17,18^ The BI6015 compound was originally identified in a high-throughput screen of compounds downregulating green fluorescence in a cell line stably expressing the *INS* promoter driving the GFP gene,^19,20^ as the *INS* promoter is well established to possess an HNF4α-binding element, and is strongly upregulated by that transcription factor. That work also showed that BI6015 downregulated HNF4α protein, and was selectively cytotoxic against Hep3B hepatocellular cancer (HCC) cells (but not primary hepatocytes). A further screen of NCI-60 cancer cells demonstrated BI6015 cytotoxicity to numerous neoplastic cell lines, but not their normal counterparts. Finally, BI6015 was efficacious in an orthotopic xenograft mouse model, in vivo, although liver stenosis was also noted, and the compound exhibited suboptimal pharmacokinetic properties.^20^

In the current study, we devised a straightforward strategy for assessing BI6015 modifications that might optimise its interactions with the compound-binding site of HNF4α, to increase specificity and druglikeness. Although previously reported studies only assessed only one derivative of BI6015, we examined movement of a nitro group, relative to a methyl group on the BI6015 benezene ring, from the *para* to the *meta* and *ortho* positions, and possible effects on specific signalling pathways important to improve pharmacokinetic properties. Our results showed that the antimitogenic activity of the parent (*para*) compound, compared to the derivatives, occurs via potent inhibition of the oncogenic signal mediator WNT. We thereby assert that design of specific transcription factors, via such chemoinformatics and bioinformatics analyses, represents a novel strategy for drug discovery. Also, the *ortho* and the meta derivatives did not inhibit HNF4α.

## Materials and methods

### General chemistry

All reactions sensitive to air or moisture were conducted under a nitrogen atmosphere. Reagents were purchased from Sigma-Aldrich (St. Louis, MO, USA) and Tokyo Chemical Industry. All the anhydrous solvents were distilled over CaH_2_, P_2_O_5_, or Na/benzophenone, prior to the reaction, unless otherwise stated. Analytical thin-layer chromatography (TLC) was performed using commercial, precoated TLC plates (silicagel 60, F-254, EMD Millipore, Burlington, MA, USA). Spots were then viewed under a ultraviolet (UV) light (254 nm), or colourising, by charring, after dipping in any of the following solutions: phosphomolybdic acid in ethanol, or potassium permanganate in aqueous solution. Flash column chromatography was performed on silica gel 60 (0.040–0.063 mm, 230–400 mesh, EMD Millipore). Infrared spectra were recorded on an Agilent (Santa Clara, CA, USA) Cary 670 Fourier-transform infrared instrument. Proton nuclear magnetic resonance (^1^H NMR) spectra (CDCl_3_, CD_3_OD, D_2_O, or dimethyl sulfoxide (DMSO)-*d*_6_) were recorded on an Agilent 400-MR (400 MHz). ^1^H NMR data were reported as peak multiplicities: s for singlet; d for doublet; dd for doublet of doublets; ddd for doublet of doublet of doublets; t for triplet; pseudo t for pseudo triplet; brs for broad singlet; and m for multiplet. Coupling constants were reported in hertz (Hz). Carbon-13 nuclear magnetic resonance (^13^C NMR) spectra (CDCl_3_, CD_3_OD, D_2_O, or DMSO-*d*_6_) were recorded on an Agilent 400-MR (100 MHz) instrument. Chemical shifts were reported as ppm (δ), relative to the solvent peak. Mass spectra were recorded on an electrospray ionization-positive source, in methylene chloride or methanol.

### General procedure for the synthesis of sulfonyl chloride (**2a** and **2b**)

**Fig. 1:**
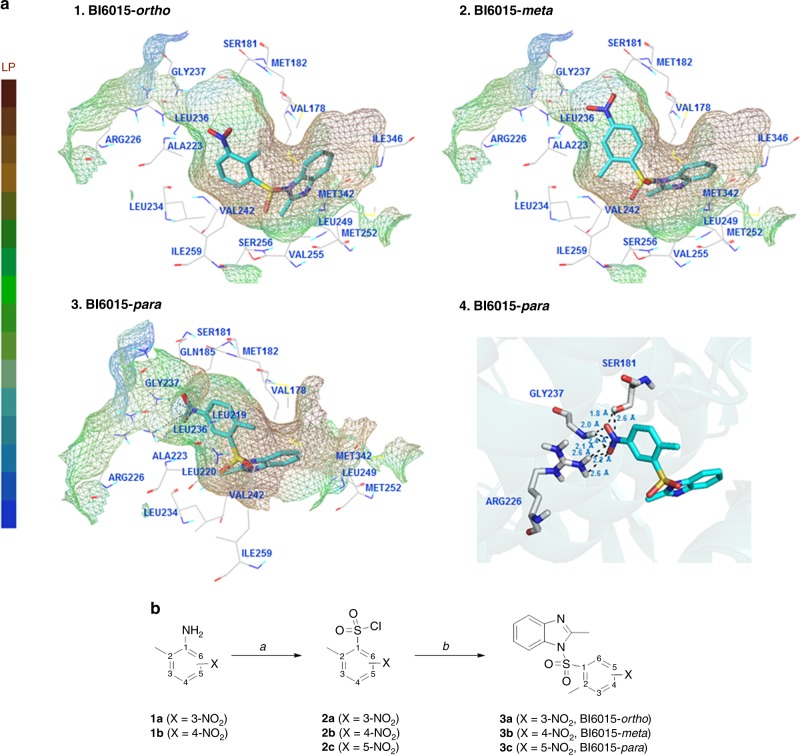
Binding modes and synthesis of BI6015 derivates. **a** The proposed binding modes of BI6015-*ortho* (**3a**), BI6015-*meta* (**3b**), and BI6015-*para* (**3c**) forms in the binding pocket of human HNF4α (PDB code 3FS1), with key amino acid residues shown. Hydrogen bonds are denoted as black dotted lines. (1) Each part of the ligand-binding pocket for the *ortho*-nitro-substituted BI6015 **3a** is represented as a lipophilic potential surface. (2) Each part of the ligand-binding pocket for the *meta*-nitro-substituted **3b** is represented as a lipophilic potential surface. (3) Each part of the HNF4α ligand-binding pocket for *para*-nitro-substituted **3c** is represented as a lipophilic potential surface. (4) The binding site for *para*-nitro-substituted **3c**, as an HNF4α ligand, is in ribbon cartoon. Amino acid residues interacting via hydrogen bonds are labeled. **b** Reagents and conditions: (a) (i) HCl, H_2_O, NaNO_2_, (ii) SOCl_2_, H_2_O, CuCl; (b) 2-methyl-1*H*-benzo[*d*]imidazole, CH_3_CN

Step (a) Thionyl chloride (4.2 mmol) was added dropwise, over 10 min, to 2-mL water, and cooled to 0 °C. The solution was then allowed to warm to 15 °C, over 16 h. Copper (I) chloride (0.01 mmol) was added to the mixture, and the resultant yellow-green solution was cooled to −3 °C. Step (b) Concentrated HCl (1 mL) was added, with stirring, to an appropriate aniline (1 mmol), using ice to maintain the temperature of the mixture below 30 °C. The reaction mixture was then cooled to −5 °C, and a solution of sodium nitrite (1.1 mmol) in water (0.3 mL) was added drop-wise, over 10 min, maintaining the temperature at −5 to 0 °C. The resultant slurry was cooled to −2 °C, and stirred for 10 min. Step (c) The slurry from step (b) was cooled to −5 °C, and added to the solution obtained from step (a), over 30 min. As the reaction proceeded, a solid began to precipitate. When the addition was complete, the reaction mixture was stirred at 0°C for 70 min, and the suspended solid collected by vacuum filtration, washed with water, and dried under vacuum, to give the corresponding sulfonyl chlorides, in 52–62% yields. The sulfonyl chloride products **2a** and **2b** were used for the next step, without further purification (Fig. 1b).

### General procedure for the synthesis of sulfonamide (**3a**, **3b**, and **3c**)

A mixture of 2-methyl-1*H*-benzo (*d*) imidazole (1 mmol), and an appropriate sulfonyl chloride compound (1 mmol) in CH_3_CN (5 mL), was stirred at room temperature for 2–5 h. Progress of the reaction was monitored by TLC. After completion of the reaction, the content was poured into ice-cold water (5 mL), while stirring. The solid was filtered, dried, and purified by recrystallisation, using MeOH or EtOH to give sulfonamide products **3a**, **3b**, and **3c**, at 63–88% yields (Fig. 1b).

### 2-Methyl-1-((2-methyl-3-nitrophenyl)sulfonyl)-1*H*-benzo[*d*]imidazole (**3a**, BI6015-*ortho*)

A white solid; IR (attenuated total reflection (ATR))cm^−1^ 1607, 1552, 1528; ^1^H NMR (400 MHz, CDCl_3_) δ 7.96 (d, *J* = 8.0 Hz, 1H), 7.84 (d, *J* = 8.0 Hz, 1H), 7.78 (d, *J* = 8.0 Hz, 1H), 7.73 (d, *J* = 8.0 Hz, 1H), 7.51 (t, *J* = 8.0 Hz, 1H), 7.38 (t, *J* = 8.0 Hz, 1H), 7.33 (t, *J* = 8.0 Hz, 1H), 2.69 (s, 3H), 2.59 (s, 3H); ^13^C NMR (100 MHz, CDCl_3_) δ 151.27, 149.67, 141.42, 140.42, 133.54, 132.50, 131.59, 129.07, 127.44, 125.23, 125.16, 120.24, 113.40, 16.98, 15.15; high-resolution mass spectrum (HRMS) calculations for C_15_H_13_N_3_O_4_S [M-H]^−^: 330.0549; found 330.0545.

### 2-Methyl-1-((2-methyl-4-nitrophenyl)sulfonyl)-1*H*-benzo[*d*]imidazole (**3b**, BI6015-*meta*)

A white solid; IR (ATR) cm^−1^ 1610, 1555, 1524; ^1^H NMR (400 MHz, CDCl_3_) δ 8.16 (m, 2H), 7.86 (d, *J* = 9.0 Hz, 1H), 7.76 (d, *J* = 8.0 Hz, 1H), 7.72 (d, *J* = 8.0 Hz, 1H), 7.35 (m, 2H), 2.71 (s, 3H), 2.60 (s, 3H); ^13^C NMR (100 MHz, CDCl_3_) δ 151.33, 150.45, 142.68, 141.45, 140.62, 133.45, 129.92, 127.86, 125.19, 125.15, 121.53, 120.22, 113.31, 20.38, 17.02; HRMS calculations for C_15_H_13_N_3_O_4_S [M-H]^−^: 330.0549; found 330.0543.

### 2-Methyl-1-((2-methyl-5-nitrophenyl)sulfonyl)-1*H*-benzo[*d*]imidazole (**3c**, BI6015-*para*)

A white solid; IR (ATR) cm^−1^ 1602, 1558, 1528; ^1^H NMR (400 MHz, CDCl_3_) δ 8.85 (d, *J* = 2.0 Hz, 1H), 8.38 (dd, *J* = 2.0, 8.0 Hz, 1H), 7.76 (d, *J* = 8.0 Hz, 1H), 7.70 (d, *J* = 8.0 Hz, 1H), 7.52 (d, *J* = 8.0 Hz, 1H), 7.34 (m, 2H), 2.72 (s, 3H), 2.52 (s, 3H); ^13^C NMR (100 MHz, CDCl_3_) δ 151.16, 146.10, 145.50, 141.48, 138.69, 134.54, 133.35, 128.54, 125.17, 125.07, 124.66, 120.23, 113.27, 20.39, 17.06; HRMS calcd for C_15_H_13_N_3_O_4_S [M + H]^+^: 332.0705; found 332.0703.

### BI6015-to-HNF4α docking studies

The structures of compounds BI6015-*ortho*, BI6015-*meta*, and BI6015-*para*, as ligands, were constructed using Sybyl-X 2.1.1 software, and energy minimised by the Powell method, using Gasteiger–Marsili charge and the Tripos force field.^21^ The crystal structure of HNF4α was obtained from the Protein Data Bank (PDB code 3FS1),^22^ and all crystal water molecules removed. Missing hydrogen atoms were added to the structures. Docking was performed using Surflex-Dock (Sybyl-X 2.1.1, Tripos Inc., St. Louis, MO, USA).^23^ For the protein, the protocol for characterising the binding site of the receptor was generated using a ligand-based approach. All other parameters accepted default settings (Fig. 1a).

AutoDock Vina (v1.1.2), a program for optimising and scoring molecular docking,^24^ was used to assess HNF4α docking to BI6015-*ortho*, BI6015-*meta*, and BI6015-*para* forms. A flexible ligand, MYR (myristic acid), was used to consider docking pose. MYR binding positions (V178, S181, Q185, R226, L236, G237, M252, S256, I259, Q345, and I346), of HNF4α, were used for the BI6015 derivatives’ docking site. The center of docking used the Cα coordinate, in each binding residue, of the receptor HNF4α. To obtain the largest number of poses, we set *num_modes* to 1000 and *energy_range* to 50. A 15Å docking box around the Cα coordinate was defined. The docked ligands, obtained by Cα docking, were then clustered using CHARMM^25^ on the center of mass (COM), and the structure with the lowest energy was selected for each cluster. The cluster radius was 4 Å. The predicted binding energy was calculated as kcal/mol, and the free energy, depending on the number of ligands in the cluster, was calculated as “lowest energy + (−*kT* ln *N*).”

### Cell culture experiments

All GC cell lines (AGS, MKN45, MKN1, SNU16, SNU668, SNU601, SNU620, NCI-N87, NCC24, NCC59, and SNU1750) were obtained from the American Type Culture Collection (Manassas, VA, USA), and grown in RPMI-1640 (Invitrogen, Carlsbad, CA, USA) and 10% foetal calf serum (Hyclone, Logan, UT, USA), at 37°C, under 5% CO_2_. Cells (2.5 × 10^5^) were seeded and grown to 70–80% confluence and treated with DMSO, 5 or 10 µM *para*-BI6015, *ortho*-BI6015, or *meta*-BI6015 for 48 h, and cell viability was determined by 3-[4,5-dimethylthiazol-2-yl]-2,5 diphenyl tetrazolium bromide (MTT) assay (Sigma). For Western blot, cells were washed twice with phosphate-buffered saline + Tween-20, lysed in 20 mM Tris (pH 7.4), 250 mM NaCl, 2 mM EDTA, and 1% Triton X-100 buffer, using total protein harvested following treatment. Each cell line was prepared at *n* = 3, using a pool of three samples per cell line prepared at 50 µg per lane, electrophoresed through sodium dodecyl sulfate-polyacrylamide gel electrophoresis gels, transferred to PVDF membranes, and treated with antibodies against HNF4α, WNT5A, or β-actin (Cell Signaling Technology).

The following human GC cell lines were used within 6 months of tissue resuscitation: NCI-N87, AGS (ATCC), MKN45 (RIKEN), SNU-484, SNU-601, and SNU-1967 (KCLB), cultured in RPMI-1640 (HyClone), and 10% foetal calf serum (HyClone) at 37 °C under 5% CO_2_. Cell line identities were validated by short tandem repeat profiling (ATCC, RIKEN, and/or KCLB).

### Reporter (luciferase) assay

Luciferase reporter assays were performed using SNU1750, AGS, MKN45, NCC24, NCC59, and NCI-N87 cells stably transfected with a TCF/LEF reporter assay system (Qiagen Sciences, Hilden, Germany). Transfected cells were then treated with 2-µM *para*-BI6015, *ortho*-BI6015, or *meta*-BI6015 (each cell line performed at *n* = 3). The positive control was a constitutively expressed green fluorescent protein (GFP) construct, and the negative control was a minimal promoter GFP reporter. Cells were lysed with passive lysis buffer, and transferred to 96-well white opaque flat-bottom plates, to assess luciferase activity via a Dual-Luciferase reporter assay system (Promega, Madison, WI, USA) and VICTOR Light (Perkin Elmer, Waltham, MA, USA). Luciferase activity was measured on days 2 and 4, post-reporter transfection.

### Statistical analysis

For individual gene experiments, *p* < 0.05 was considered statistically significant, based on Student's *t*- or *z* tests, when comparing two groups (replicates *n* = 3 per group) (Fig. 4).

### Gene expression assessments and analysis

Following the above-mentioned drug treatments (AGS, SNU216, SNU601, SNU668, and MKN1 at 10-µM *para*-BI6015, *ortho*-BI6015, or *meta*-BI6015 for 48 h; each cell line performed at *n* = 3), total cellular messenger RNA (RNA) was isolated, using RNeasy kits (Qiagen), reverse transcribed, and hybridized to gene expression microarrays (Thermo Fisher Scientific), using a procedure we published previously.^17^ Gene expression was then assessed by unsupervised hierarchical clustering, with results visualised using TreeView.^26^ Gene set enrichment analysis (GSEA)^27^ was then used for pathway analysis of the gene expression results.

### Hierarchical clustering and gene set enrichment analysis

Gene expression profiles were stratified by unsupervised hierarchical clustering, with results depicted using R. GSEA^27^ was then used for pathway analysis of the genes found significantly misexpressed.

### Subpathway analysis

Subpathway analyses were performed using our published algorithm, PATHOME,^28^ designed to statistically discover differentially expressed subpathways, using KEGG (Kyoto Encyclopedia of Genes and Genomes)^29^ as its prior knowledge of pathway structure. This algorithm requires gene expression levels of both control vs. case groups. Therefore, we performed subpathway analysis on each of the three *para*-, *ortho*-, and *meta*-treated cell line (AGS, SNU216, SNU601, SNU668, and MKN1) datasets, as the case group, and the DMSO-treated cell lines, as the control group. The statistical significance cut-off was set to 0.05. After that, we distinguished genes that were commonly or uniquely identified, in each subpathway analysis, by merging the results of each subpathway analysis, based on their unique symbols. Because the interaction types (e.g., inhibition, activation), between two neighboring genes, were conserved (as registered in KEGG), we exported the merged results to Cytoscape,^30^ to generate a network diagram. Among commonly identified genes, we performed a statistical test, one-way analysis of variance (ANOVA), with *p* value cut-offs set to 0.05, to identify genes differentially dysregulated for each treatment group.

### Survival analysis

#### Clinical dataset

Clinical censoring data (including race), for survival analysis, was obtained from a The Cancer Genome Atlas (TCGA)^31^ stomach adenocarcinoma (STAD) (i.e., TCGA STAD) gene expression database (originally determined by RNA-sequencing (IlluminaHiseq, version: 2015-02-24)), downloaded from the UCSC Cancer Genomics Browser,^32,31^ in cBio Portal.^33^ The number of live, deceased, or absent patient samples, available for overall survival (OS) and gene mRNA expression, was 368.

For each gene previously found statistically significant by ANOVA, we calculated the 1st (bottom 25%) and 3rd (upper 25%) quantile values, from the 368 samples. These quantiles were then used for dividing the TCGA Asian samples (77 samples) into their respective bottom and upper 25% groups. The mRNA expression level of the bottom 25% was less than the 1st quantile value, and that of upper 25% was greater than the 3rd quantile value. We then compared the two groups,^34^ using a log-rank test (Fig. 6).

## Results

### Protein–ligand interaction modelling for predicting mode of binding inhibition

Molecular docking studies were performed to extensively probe structure–activity relationships, and possible binding modes, of the three BI6015 derivatives, within the HNF4α ligand-binding pocket. Specifically, we hypothesised that relocation of a nitro group, from the *para* position, relative to a methyl group on the BI6015 benzene ring, to the *ortho* and *meta* positions, could alter HNF4α downstream signalling, and possibly increase ADME (i.e., Absorption, Distribution, Metabolism, and Excretion) properties of the drug.^35,36^ These BI6015 derivatives, as ligands, were docked into the ligand-binding pocket of human HNF4α (PDB code 3FS1)^21^ as a receptor, using Surlflex-Dock (SYBYL-X 2.1.1) (Fig. 1).

A binding pose comparison of the BI6015-*ortho*, BI6015-*meta*, and BI6015-*para* compounds, within the ligand-binding pocket of human HNF4α, is shown in Fig. 1a. The protein ligand-binding site is represented by Connolly surface amino acids, colour-coded according to lipophilicity. The brown areas are lipophilic, the blue areas hydrophilic, and the green areas neutral. Side chains of the key amino acid residues, which may participate in hydrogen bonding with the BI6015 derivatives, are shown. Hydrogen bonds between the ligand (i.e., the BI6015 derivatives) and the side chains of HNF4α amino acids are represented by black-dotted lines.

Analysis of BI6015 docking revealed that its phenyl ring-bound nitro group should be buried, to accept hydrogen bonds from the HNF4α ligand-binding domain, on its lipophilic surface. These results showed that the *ortho*-nitro and *meta*-nitro substitutions caused a steric clash that interfered with BI6015-*ortho* and BI6015-*meta* derivative binding, whereas the *para*-nitro (parent compound) allowed interaction of the nitro group, via strong hydrogen bonding with HNF4α. The correlation in ribbon cartoon between substituted BI6015-*para* and the key amino acid residues in the HNF4α-binding site is depicted in Fig. 1a, showing an extensive hydrogen bonding interaction of the nitro group of BI6015-*para* and the amine or hydroxyl groups of three amino acid residues (SER181, ARG226, and GLY237), within the ligand-binding pocket of human HNF4α.

**Fig. 2:**
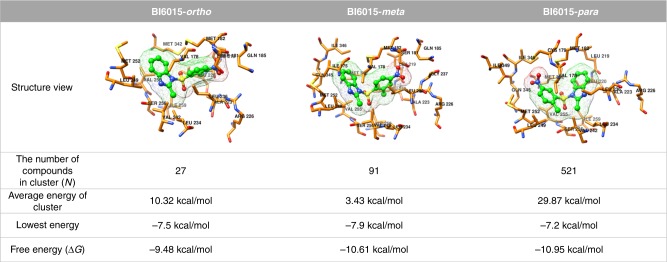
Docking results for BI6015-*ortho*, BI6015-*meta*, and BI6015-*para* forms. The BI6015-*ortho*, BI6015-*meta*, and BI6015-*para* forms are represented by green “balls and sticks,” and a mesh surface. The residues of HNF4α are indicated by an orange stick. HNF4α residue numbers are shown in black. The number of compounds in each cluster represents the number of similarly posed, docked BI6015 derivatives with the lowest energies. All structures were drawn using the Chimera software

We also found that the BI6015-*para* derivative had the most energy-stable structure. Figure 2 shows the docking energy results for BI6015 derivative binding to the HNF4α ligand-binding pocket, as determined using Autodock Vina 1.1.2. That analysis successfully identified numerous docking results as changes in the poses of BI6015 (see “BI6015-to-HNF4α Docking Studies,” in the Materials and methods). Docked compounds were clustered according to similar poses and positions, and the clusters having the lowest binding energies then selected. For each structure, BI6015 derivatives are shown in the form of green balls-and-sticks, and their HNF4α-interacting residues represented by orange sticks (Fig. 2).

In Fig. 2, “The number of compounds in cluster” is the final number of ligands in the selected cluster groups, and the number of docked BI6015-*ortho*, BI6015-*meta*, and BI6015-*para* derivatives were 27, 91, and 521, respectively, showing the BI6015-*para* cluster group to have the most similar poses within the binding pocket. The average energy of the clusters was 10.32 kcal/mol for the *ortho* form, 3.43 kcal/mol for the *meta* form, and 29.87 kcal/mol for the *para* form. However, the *ortho* form showed a large energy deviation between the BI6015 ligands forming the cluster, while the energy deviation of the *meta* form ligands was small. The average energy of the docked para form was relatively higher than the others, because of the large number (521) of ligands forming the cluster, and the fact that their distribution did not converge to the binding site. The ligand with the lowest binding energy was BI6015-*meta* at −7.9 kcal/mol, and thus could be judged as more structurally stable than the other forms. However, when the free energy, considering entropy, was measured, the BI6015-*para* form had the lowest free energy, −10.95 kcal/mol, indicating it to be the energy-stable, at the HNF4α ligand-binding site, of the three derivatives, thus forming stronger bonds.

### Chemistry

For the mechanistic study of BI6015 derivatives binding to the pocket of HNF4α, we focused on the design and synthesis of structurally related BI6015 derivatives, in which a nitro (NO_2_) group is, respectively, relocated to the *ortho* (**3a**) *meta* (**3b**) positions, as opposed to the parent compound, with the nitro group at the *para* position (**3c**, BI6015),^37^ relative to a methyl group, on the benzene ring (Fig. 1b).

2-Methyl-3-nitrobenzenesulfonyl chloride (**2a**) and 2-methyl-4-nitrobenzenesulfonyl chloride (**2b**) were synthesized from commercially available 2-methyl-3-nitro aniline and 2-methyl-4-nitro aniline, respectively, using aqueous acidic conditions, via a diazonium ion intermediate, in the presence of copper salts, with thionyl chloride as the sulfur dioxide (SO_2_) source. After conversion of the amine group to sulfonyl chloride, on the phenyl ring, the treatment of an appropriate sulfonyl chloride (**2a** or **2b**) with 2-methyl-1*H*-benzo[*d*]imidazole, in acetonitrile, afforded the desired 3-NO_2_ (**3a, BI6015-*ortho***) or 4-NO_2_ (**3b, BI6015-*meta***) derivative. Also, treatment of 2-methyl-1*H*-benzo[d]imidazole with commercially available 2-methyl-5-nitrobenzenesulfonyl chloride (**2c**), in acetonitrile, gave the parent compound (**3c, BI6015-*para***).

### Surface plasmon resonance: protein–ligand interactions

**Fig. 3:**
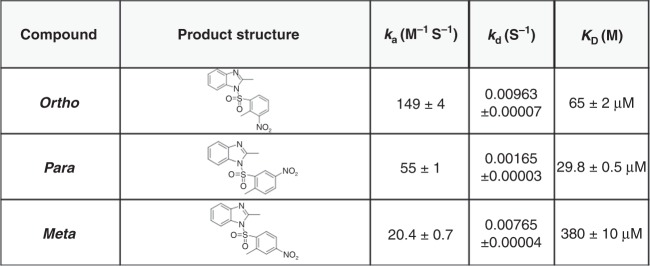
BI6015-*ortho*, BI6015-*meta*, and BI6015-*para* *K*_D_ values, as determined by surface plasmon resonance

We next performed surface plasmon resonance (SPR) to analyse protein–ligand interactions, and kinetic constants, between HNF4α and the BI6015 derivatives, including determination of the on-rate (*k*_a_), off-rate (*k*_d_), and dissociation constant (*K*_D_). Of the three compounds, BI6015-*para* had the lowest *k*_d_ and *K*_D_ values (0.00165 and 29.8μM, respectively), compared to BI6015-*meta* and BI6015-*ortho* (Fig. 3), and would thus be predicted to most affect HNF4α and its downstream signalling.

#### Experimental validation of the three (BI6015-*para* and its two derivative) compounds

**Fig. 4:**
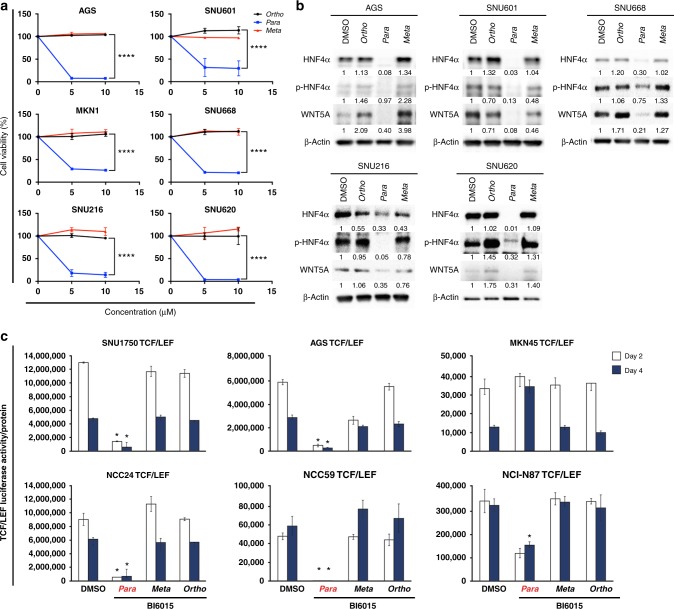
BI6015 inhibition of gastric cancer (GC) cell growth and WNT signalling. **a** Cell viability assays were performed in the treatment of BI6015. Six cell lines were selected based on their HNF4α and RhoA protein expression levels, AGS (mid-level RhoA expression), SNU601, SNU668, SNU216, and SNU620 (high-level expression), and MKN1 (low-level expression). The cell lines were treated with 5 and 10μM of the three BI6015 derivatives. **b** Western blot analysis showing HNF4α and WNT5A protein levels, and the five GC cell lines were treated with the three BI6015 derivatives for 48 h. **c** Monitoring of WNT signalling activity, using a TCF/LEF-*luc* reporter luciferase assay, followed by 2µM of the three BI6015 derivatives, for 48 or 96h, in six GC cell lines (SNU1750-, AGS-, MKN45-, NCC24-, NCC59-, NCI-N87-TCF/LEF). Because of the cell viability within the GC cell line panel,^17^ few cell lines failed to meet the transfection quality to perform TCF/LEF reporter assay. Therefore, we showed different cell lines to explain the study (*p <0.05 and ****p <0.005) (error bar: the standard error of the mean)

For validating the in silico and SPR findings described above, we treated six diverse GC cell lines with parental BI6015 (*para*-nitro), and its *ortho* and *meta* derivatives. Using 96-well plates, 2 × 10^5^ cells were treated for 48 h with 5 or 10 µM of each BI6015 derivative, and then the viability was determined by MTT assay. As shown in Fig. 4a, in agreement with predicted and measured binding properties, the parent compound (*para* nitro) caused the greatest loss of viability, at nearly 100%, of SNU620 and AGS GC cells, at ~90%, of SNU216 cells, but slightly less so (70–75%), of the remaining three GC lines (SNU601, SNU668, and MKN1). These assays demonstrated the potency of this compound. Although not shown here, we previously demonstrated negligible cytotoxicity of BI6015-*para* toward normal gastric mucous columnar cells,^17^ similar to another report of BI6015 treatment of numerous normal epithelial cell lines.^20^

Since BI6015 was previously demonstrated as antitumorigenic against HCC, largely via its destabilising effects on HNF4α,^20^ we next examined parental (*para*) BI6015, and its *meta* and *ortho* analogs for such properties, against five GC cells, AGS (mid-level HNF4α expression), and SNU601, SNU668, SNU216, and SNU620 (low HNF4α expression). These results largely supported the cell viability assays of Fig. 4a, showing that the cells most sensitive to BI6015 had the greatest losses of HNF4α (Fig. 4b and SI, Figure S1). Analogously, those with less endogenous HNF4α (SNU601, MKN1, SNU216, and SNU668, Fig. 4 and Supplementary Figure S1), despite further protein loss, were less growth inhibited. However, loss of WNT5A protein expression was greater by *para*-BI6015, in comparison to its *meta* and *ortho* analogs (Fig. 4b), thus showing downregulation of not only HNF4α but also its downstream target genes, including *WNT5A*.

#### In vitro inhibition of WNT signalling by BI6015-*para*, and its two derivative compounds

To validate our previous findings of the importance of a WNT5A/HNF4α signalling cascade, to GC progression,^17^ we stably transfected GC cell lines (AGS, SNU1750, MKN45, NCC24, NCI-N87, and NCC59) with a TCF/LEF-*luc* reporter or empty vector, to quantify possible WNT pathway downregulation. Specifically, selection of these GC cell lines was based on low-to-high RhoA protein expression levels, and an established WNT pathway-responsive reporter system.^38^ Each GC cell line was treated with 2-µM BI6015, and its derivatives, and luciferase activity subsequently measured on days 2 and 4, post-reporter transfection. As shown in Fig. 4c, 60% (NCI-N87) to 95% (AGS, SNU1750, NCC24) to 100% (NCC59) luciferase activity was inhibited in five of the six GC cell lines, using the parental *para*-BI6015. However, in the treatment of the *ortho* and *meta* compounds (Fig. 4b, c), none of these five cells demonstrated significant WNT inhibition, or WNT5A downregulation. One GC cell line, MKN45, underwent no drug inhibition (by any BI6015 analog) of WNT signalling, again demonstrating the challenges in drug development for this highly heterogeneous malignancy.^39^ These data do, however, validate our previous finding that the TCF/LEF reporter assay system is a sensitive HNF4α drug readout, as shown in Fig. 4b, and its downstream pathway, in cross-talk with other HNF4α targets (Figs. 4b, c). While WNT5A was one HNF4α target downregulated by *para*-BI6015, we cannot conclude, however, that loss of TCF/LEF reporter activity was strictly due to loss of WNT5A, as this assay merely measures total WNT activity, and cannot discriminate between specific WNT isoforms.

#### Parental BI6015 (-*para*) treatment induces differential gene dysregulation, in inhibiting the WNT signalling pathway

**Fig. 5:**
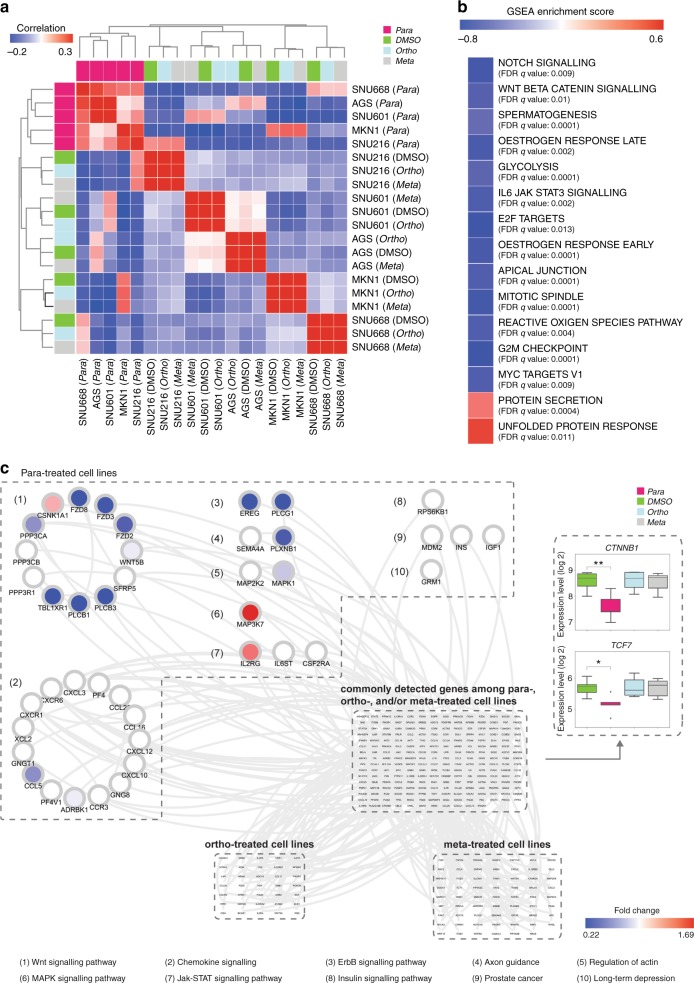
*Para*-treated cell lines showed similarly dysregulate gastric cancef (GC) cell line responses at the molecular level. **a** Transcriptome correlation clustering analysis showed exclusive clustering of the parental *para*-treated, but not dimethyl sulfoxide (DMSO)-treated, *meta*-treated, or *ortho*-treated, cell lines, with the latter four clustering only by replicates of individual cell types. As shown, the *para*-treated cell lines had similarly dysregulated gene expression levels. **b** Based on the results of the correlation clustering analysis, GSEA analysis was performed, by setting the cell lines treated with *para*-BI6015 vs. *meta*-BI6015 and *ortho*-BI6015 as case groups and control groups, respectively. The top 15 gene sets significantly associated with gene expression differences between case and control groups are shown, specifically revealing the WNT signal pathway. **c** We merged the three subpathway analyses by our network analysis algorithm, PATHOME,^28^ from *ortho*- vs. DMSO-treated, vs. *meta*- vs. DMSO-treated, vs. *para*- and DMSO-treated cell lines. In the merged subpathway networks, we found not only commonly detected subpathway genes from all three analyses but also treatment-specific subpathway genes. Significantly dysregulated subpathways, including WNT signal pathway genes, uniquely detected in *para*-BI6015- vs. DMSO-treated cells, are enclosed in the upper left dashed box. Subpathways uniquely detected in other analyses, including *ortho*- vs. DMSO-treated cells, and *meta*- vs. DMSO-treated cells, are indicated by the dashed boxes shown at the bottom. Subpathways commonly detected in all three analysis results are shown enclosed in the dashed box in the middle. *CTNNB1* and *TCF7* were detected as statistically significant in all three *para*-BI6015-, *ortho*-BI6015-, and *meta*-BI6015-treated cell lines; however, they were significantly downregulated only by the *para*-compound. Positive GSEA scores (red colour) meant that a gene set was enriched in *para*-compound-treated cell lines, while a negative GSEA score (blue colour) meant that a gene set was enriched in cell lines treated with the other BI6015 derivatives (*p -value <0.05, **p -value <0.01)

We next performed correlation clustering analysis of BI6015-treated GC cells, based on gene expression microarray results. As shown in Fig. 5a, cells treated with the parental (*para* position) compound correlated, at the molecular level, into one cluster (left columns, Fig. 5a). However, treatment with vehicle (DMSO), or the two substituents (*ortho* and *meta* nitro derivatives), did not change cell-to-cell transcriptomes, and thus these cells merely clustered among themselves, irrespective of drug treatment (DMSO or *meta* or *ortho* compounds).

Since clustering analysis showed that the *para*-treated cell lines correlated at the molecular level, we then compared these cells with those treated with the other BI6015 derivatives, using GSEA, with hallmark gene sets from MSigDB. GSEA showed that *para*-compound-treated cell lines effectively downregulated the WNT, Notch, and other oncogenic signalling pathways (Fig. 5b), compared to GC cell lines treated with the other BI6015 derivatives.

With clustering and GSEA analyses, we found that *para*-BI6015-treated cell lines correlated in gene expression, and various functional contexts, including suppression of the WNT and Notch embryonic signalling pathways. We then performed subpathway analysis to identify distinctly dysregulated signalling subpathways and genes, using our previously developed tool, PATHOME,^28^ which enables delineation of statistically significant differential expression patterns, along specific subpathways. We then merged the resulting differentially expressed subpathways, of the three experimental groups (AGS, MKN-1, and SNU601 GC cell lines treated with 10-µM BI6015 *para*, *ortho*, and *meta* derivatives), compared to the DMSO-treated control group, to distinguish commonly vs. uniquely dysregulated genes. We first sought gene expression patterns, commonly identified among cell lines treated with each specific compound, by performing a statistical test, one-way ANOVA.

Based on the above determinations, 23 genes were found as commonly differentially expressed among the *para*-treated GC cell lines, compared to the other cell lines (*p* < 0.05, Supplementary Figure S2 and Supplementary Table S1). Among these 23 genes, in *para*-treated cell lines, *CTNNB1* and *TCF7*, members of the WNT signalling pathway (Supplementary Table S2), showed significantly dysregulated (mostly downregulated) patterns, compared to the DMSO-treated and the *ortho*-BI6015- and *meta*-BI6015-treated cell lines. Then, we sought dysregulated patterns among genes belonging to subpathways found statistically significant by PATHOME, to identify which genes and subpathways were affected, with merged subpathways presented as a network diagram (Fig. 5c; for all uniquely identified genes of each treatment group, see Supplementary Figure S3).

Uniquely discovered genes were then arranged in the order of *para*-BI6015-, *meta*-BI6015-, and *ortho*-BI6015-treated cell lines (clockwise from the upper left in Fig. 5c), with commonly identified genes arranged in the center of the network. The *para* (parental BI6015)-treated cell lines showed more dysregulated patterns of genes than the *ortho*-BI6015- and *meta*-BI6015-treated cell lines (Supplementary Figure S3). In particular, the WNT signalling pathway of the *para*-BI6015-treated cell lines had a greater number of dysregulated (primarily downregulated) genes (Fig. 5c, marked as circle # 1). Based on this network analysis, the WNT signalling pathway of the *para*-BI6015-treated cell lines showed highly dysregulated gene expression patterns, in accord with our previous reports,^17,18^ showing that the HNF4α/WNT signalling pathway plays an important role in GC, thus representing a strong therapeutic target.

**Fig. 6:**
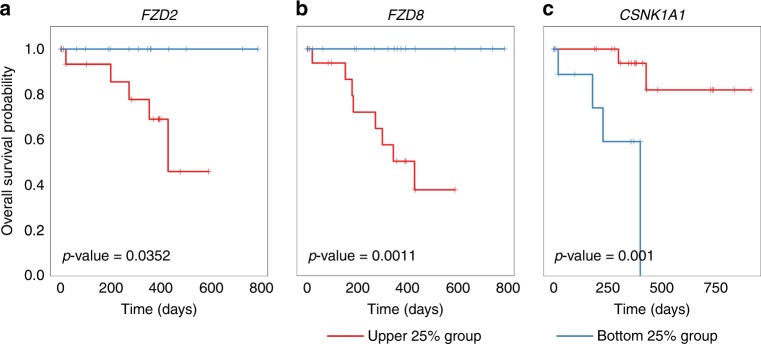
*FZD2*, *FZD8*, and *CSNK1A1*, WNT signal pathway genes, were dysregulated by *para*-BI6015-treated gastric cancer (GC) cancer cell lines, but divergently influenced patient survival, assessed using a TCGA STAD Asian dataset. The total available number of samples that had survival information and mRNA expression levels in a TCGA STAD dataset was 368. We calculated the 1st quantile and the 3rd quantile of gene expression levels. By using these quantiles for dividing Asian samples (*n* = 77) in the TCGA dataset into two groups, and upper 25% and bottom 25% group (see details in the “Survival analysis” section of the Methods). For *FZD2*, the number of samples in the bottom 25% group was 16 and that in upper 25% group was 23. For *FZD8*, the sample numbers in the upper 25% and bottom 25% groups were 24 and 20, respectively. For *CSNK1A1*, the sample numbers in the upper 25% and bottom 25% groups were 22 and 14, respectively. “+” marks show censored patients. *P* values of survival analysis were derived by the log-rank test

In addition, using The Cancer Genome Atlas STomach ADenocarcinoma (TCGA-STAD) Asian patient dataset, we performed survival analysis for each gene found differentially expressed, in the *para*-BI6015-treated group, compared to the other groups (for group stratification criteria, see the Materials and methods). As shown in Fig. 6, the WNT pathway genes *FZD2*, *FZD8*, and *CSNK1A1* were found significant to GC OS, as determined by log-rank test, with *p* values of 0.0352, 0.0011, and 0.001, respectively.

## Discussion

In this study, we extensively characterised the efficacy and pharmacology of BI6015, an inhibitor of the transcription factor, HNF4α, a “master” signal mediator we^17,18^ and others^40,41^ previously showed to be downregulated by the metabolic sensor, AMPK. HNF4α is most highly expressed in the liver, kidney, intestine, and pancreas, where its dysregulation is strongly linked to type 2 diabetes, due to its downregulation of the *INS* promoter, via binding to an alternative promoter.^42^ By exploiting that relationship, using an *INS* reporter in a high-throughput compound screen,^19^ an HNF4α inhibitor, BI6015, was discovered; BI6015 was also selectively cytotoxic to numerous cancer (but not normal) cells. However, while an in vivo study of BI6015 in murine liver cancer showed antitumour efficacy, shortcomings included liver stenosis and suboptimal bioavailability.^20^

Previous reports indicate that two Wnt genes, *FZD2* and *FZD8*, play roles in the metastasis-promoting epithelial–mesenchymal transition.^43,44^ Moreover, *CSNK1A1* is recognised as a tumour suppressor gene, and its dysregulated gene expression-associated transcriptome very likely drives cancer progression.^45,46^ Considering those reports, downregulation of *FZD2* and *FZD8*, in parallel with upregulation of *CSNK1A1*, might favor survival in GC patients; thus, these genes represent potential GC biomarkers. These findings concur with our results in BI6015-*para*-treated cancer cell lines, showing downregulation of *FZD2* and *FZD8*, and *CSNK1A1* upregulation (Fig. 5c), also supporting our survival analysis result (Fig. 6).

Here, based on the above-described findings, in an attempt to understand the potential efficacy of BI6015, we relocated a nitro group on its benzene ring, from the *para* position (with regard to a methyl group) to the *meta* and *ortho* positions (Fig. 3). We demonstrated that the *para* compound (i.e., the parental BI6015) most optimally bound the HNF4α ligand-binding site, via two polar (SER181 and ARG226) and one apolar (GLY237) amino acid residues (Figs. 1a, 2). We also demonstrated that *para*-BI6015 had the lowest free energy, and the greatest number of ligand structures in its lowest energy cluster, compared to the *ortho*-BI6015 and meta-BI6015 derivatives (Figs. 1a, 2). Therefore, this result means that BI6015-*para* can play a role in inhibiting binding better than the myristic acid, which originally binds HNF4α.^20^ SPR further showed the parent compound (*para* position) to possess the lowest off-rate (*k*_d_), dissociation rate (*K*_D_), and highest on-rate (*k*_a_), compared to the *meta* and *ortho* derivatives (Fig. 3).

In addition to AMPK downregulation of HNF4α, we also reported that this event coincided with repression of the HNF4α target gene, *WNT5A*,^17,18,28^ in accord with earlier findings of small interfering RNA effects on HNF4α target genes in hamster kidney and ovarian cells.^40^ By examining the transcriptomes of *ortho*-, *meta*-BI6015-, or *para*-BI6015-treated cells, we found 23 genes specifically dysregulated by BI6015 (*para*), but not its *ortho* or *meta* derivatives. We then validated downregulation of WNT activity, as assessed by reporter assays, demonstrating 60 to ~100% inhibition, in 5/6 GC cell lines. Since WNT is a well-known cancer stem cell pathway, its downregulation (i.e., by reduced HNF4α) should also be GC antitumorigenic, and we demonstrated substantial (75 – 95%) losses of cell viability, and of six diverse GC cell lines, by the parent (*para*) compound (but not the *ortho*-derivatives and *meta*-derivatives) (Fig. 4); we also observed *para*-BI6150 downregulation of HNF4α in those same cell lines, analogous to a previous study of HepG2 cells.^20^ However, we concede that optimal binding of the *para*-BI6015 compound, to HNF4α alone, cannot fully explain the antitumour efficacy of the compound, requiring further studies such as subpathway analysis.

It has been contended that subpathway analysis, used to identify highly important “hub” genes within associated networks, might be more biologically meaningful than identifying mere gene sets^47^ or pathway cross-talk. Consequently, we used our PATHOME algorithm to identify distinct *para*-BI6015-associated subpathways, identifying in particular, the WNT pathway. That finding was in agreement with our current (Fig. 4) and previous^17,18,28^ findings. In future studies, we wish to even more extensively use subpathway analyses, to identify specific mechanisms of HNF4α, and WNT signalling, dysregulation, in GC.

Our top-down, in silico approach used in this study may be applicable to identify modes of mechanism in drug development for other cancer types. Since we revealed that a specific chemical structure disturbs specific cancer cell transcriptomes, in contrast to other materials (i.e., vehicle control or alternate structures), and showed distinct perturbations in gene expression, using clustering analysis. At that juncture, we could narrow down aberrant signalling to several GC cell functional contexts (e.g., WNT and Notch signalling pathways) that were dysregulated, based on a functional gene set analysis, GSEA. Moreover, our recent meta-analysis^48^ indicated that WNT5A, belonging to WNT signalling, had positive correlations with lymph node metastasis statuses and tumour depth T stages. Also, high WNT5A expression was significantly associated with Lauren diffused scattered type.^48^ Considering these clinical significances in GC, WNT5A has potential to be developed toward a therapeutic biomarker candidate. Using a subpathway analysis tool, PATHOME, we sought not only perturbed genes but also subpathways impacted by our drug candidate compound, likely expanding our understanding of a possible mode(s) of mechanism. This approach is significantly time-saving and cost-saving, for drug development, in contrast to in vitro and in vivo preclinical strategies.

In conclusion, we have outlined a means of examining specific compounds, biochemically and biologically, for their ability to inhibit the activity of transcription factors, a pursuit that has remained challenging for some time.^14–16^ Although receptor tyrosine kinase inhibitors have demonstrated limited clinical success against gastrointestinal cancers, strategies to target additional disease-implicated proteins (e.g., transcription factors) as mono-therapies or multi-therapies represent an additional promising strategy. Finally, this work firmly establishes the role of one particular transcription factor, HNF4α, in GC etiology and progression, supporting our previous extensive work. Targeting either this protein directly or its network-associated genes represents an encouraging approach against this still prevalent and lethal disease. In a broader perspective, we believe that these chemoinformatic and bioinformatic approaches will strongly facilitate the design of “druggable” transcription factor inhibitors, targeting specific oncogenic pathways, in gastric and other liquid and solid tumours.

## Supplementary information

Supplementary Material

## Data Availability

GEO accession GSE114626 deposited.

## References

[1] Siegel RL, Miller KD, Jemal A (2017). Cancer statistics, 2017. CA Cancer J. Clin..

[2] Torre LA, Siegel RL, Ward EM, Jemal A (2016). Global cancer incidence and mortality rates and trends—an update. Cancer Epidemiol. Biomark. Prev..

[3] Bollschweiler E, Berlth F, Baltin C, Monig S, Holscher AH (2014). Treatment of early gastric cancer in the Western World. World J. Gastroenterol..

[4] Cristescu R (2015). Molecular analysis of gastric cancer identifies subtypes associated with distinct clinical outcomes. Nat. Med..

[5] Bang YJ (2010). Trastuzumab in combination with chemotherapy versus chemotherapy alone for treatment of HER2-positive advanced gastric or gastro-oesophageal junction cancer (ToGA): a phase 3, open-label, randomised controlled trial. Lancet.

[6] Iqbal S (2011). Southwest Oncology Group study S0413: a phase II trial of lapatinib (GW572016) as first-line therapy in patients with advanced or metastatic gastric cancer. Ann. Oncol..

[7] Lordick F (2013). Capecitabine and cisplatin with or without cetuximab for patients with previously untreated advanced gastric cancer (EXPAND): a randomised, open-label phase 3 trial. Lancet Oncol..

[8] Zhan T, Rindtorff N, Boutros M (2017). Wnt signaling in cancer. Oncogene.

[9] Polk DB, Peek RM (2010). *Helicobacter pylori*: gastric cancer and beyond. Nat. Rev. Cancer.

[10] Takahashi-Yanaga F, Kahn M (2010). Targeting Wnt signaling: can we safely eradicate cancer stem cells?. Clin. Cancer Res..

[11] Takebe N (2015). Targeting Notch, Hedgehog, and Wnt pathways in cancer stem cells: clinical update. Nat. Rev. Clin. Oncol..

[12] Anastas JN (2015). Functional crosstalk between WNT signaling and tyrosine kinase signaling in cancer. Semin. Oncol..

[13] Song L, Li ZY, Liu WP, Zhao MR (2015). Crosstalk between Wnt/beta-catenin and Hedgehog/Gli signaling pathways in colon cancer and implications for therapy. Cancer Biol. Ther..

[14] Darnell JE (2002). Transcription factors as targets for cancer therapy. Nat. Rev. Cancer.

[15] Dunker AK, Uversky VN (2010). Drugs for ‘protein clouds’: targeting intrinsically disordered transcription factors. Curr. Opin. Pharmacol..

[16] Yeh JE, Toniolo PA, Frank DA (2013). Targeting transcription factors: promising new strategies for cancer therapy. Curr. Opin. Oncol..

[17] Chang HR (2016). HNF4alpha is a therapeutic target that links AMPK to WNT signalling in early-stage gastric cancer. Gut.

[18] Kim YH (2012). AMPKalpha modulation in cancer progression: multilayer integrative analysis of the whole transcriptome in Asian gastric cancer. Cancer Res..

[19] Kiselyuk A (2010). Phenothiazine neuroleptics signal to the human insulin promoter as revealed by a novel high-throughput screen. J. Biomol. Screen..

[20] Kiselyuk A (2012). HNF4alpha antagonists discovered by a high-throughput screen for modulators of the human insulin promoter. Chem. Biol..

[21] Moyna G, Hernandez G, Williams HJ, Nachman RJ, Scott AI (1997). Development of Weiner et al. force field parameters suitable for conformational studies of [1,4]-benzodiazepines and related compounds. J. Chem. Inf. Comput. Sci..

[22] Rha GB, Wu G, Shoelson SE, Chi YI (2009). Multiple binding modes between HNF4alpha and the LXXLL motifs of PGC-1alpha lead to full activation. J. Biol. Chem..

[23] Spitzer R, Jain AN (2012). Surflex-Dock: docking benchmarks and real-world application. J. Comput. Aided Mol. Des..

[24] Trott O, Olson AJ (2010). AutoDock Vina: improving the speed and accuracy of docking with a new scoring function, efficient optimization, and multithreading. J. Comput. Chem..

[25] Brooks BR (2009). CHARMM: the biomolecular simulation program. J. Comput. Chem..

[26] Saldanha AJ (2004). Java Treeview—extensible visualization of microarray data. Bioinformatics.

[27] Hung JH, Yang TH, Hu Z, Weng Z, DeLisi C (2012). Gene set enrichment analysis: performance evaluation and usage guidelines. Brief Bioinform..

[28] Nam S (2014). PATHOME: an algorithm for accurately detecting differentially expressed subpathways. Oncogene.

[29] Kanehisa M, Goto S (2000). KEGG: Kyoto Encyclopedia of Genes and Genomes. Nucleic Acids Res..

[30] Shannon P (2003). Cytoscape: a software environment for integrated models of biomolecular interaction networks. Genome Res..

[31] Cancer Genome Atlas Research N. (2014). Comprehensive molecular characterization of gastric adenocarcinoma. Nature.

[32] Zhu J (2009). The UCSC Cancer Genomics Browser. Nat. Methods.

[33] Cerami E (2012). The cBio cancer genomics portal: an open platform for exploring multidimensional cancer genomics data. Cancer Discov..

[34] Mantel N (1966). Evaluation of survival data and two new rank order statistics arising in its consideration. Cancer Chemother. Rep..

[35] Lipinski CA (2000). Drug-like properties and the causes of poor solubility and poor permeability. J. Pharmacol. Toxicol. Methods.

[36] Yalkowsky SH, Valvani SC (1980). Solubility and partitioning I: solubility of nonelectrolytes in water. J. Pharm. Sci..

[37] Alexander SP (2017). The concise guide to pharmacology 2017/18: nuclear hormone receptors. Br. J. Pharmacol..

[38] Chang HR (2016). Systematic approach identifies RHOA as a potential biomarker therapeutic target for Asian gastric cancer. Oncotarget.

[39] Lim B, Kim JH, Kim M, Kim SY (2016). Genomic and epigenomic heterogeneity in molecular subtypes of gastric cancer. World J. Gastroenterol..

[40] Hong YH, Varanasi US, Yang W, Leff T (2003). AMP-activated protein kinase regulates HNF4alpha transcriptional activity by inhibiting dimer formation and decreasing protein stability. J. Biol. Chem..

[41] Leclerc I (2001). Hepatocyte nuclear factor-4alpha involved in type 1 maturity-onset diabetes of the young is a novel target of AMP-activated protein kinase. Diabetes.

[42] Miura A (2006). Hepatocyte nuclear factor-4alpha is essential for glucose-stimulated insulin secretion by pancreatic beta-cells. J. Biol. Chem..

[43] Bian Y (2016). Promotion of epithelial–mesenchymal transition by Frizzled2 is involved in the metastasis of endometrial cancer. Oncol. Rep..

[44] Gupta S (2010). FZD4 as a mediator of ERG oncogene-induced WNT signaling and epithelial-to-mesenchymal transition in human prostate cancer cells. Cancer Res..

[45] Schneider RK (2014). Role of casein kinase 1A1 in the biology and targeted therapy of del(5q) MDS. Cancer Cell.

[46] Sinnberg T, Wang J, Sauer B, Schittek B (2016). Casein kinase 1alpha has a non-redundant and dominant role within the CK1 family in melanoma progression. BMC Cancer.

[47] Li X, Shen L, Shang X, Liu W (2015). Subpathway analysis based on signaling-pathway impact analysis of signaling pathway. PLoS ONE.

[48] Nam S, Chung JW, Yang JY (2017). WNT5A correlates with clinicopathological characteristics in gastric cancer: a meta-analysis. Cell. Physiol. Biochem..

