# Supplementary material for: Differential effects, on oncogenic pathway signalling, by derivatives of the HNF4 α inhibitor BI6015
Source: Br J Cancer. 2019 Feb 22;120(5):488–98. doi: 10.1038/s41416-018-0374-5 (PMC6461897; doi:10.1038/s41416-018-0374-5)
Supplement: Supplementary file 1 — Supplementary Material [file 41416_2018_374_MOESM1_ESM.pdf]

Supporting Information, Figure S1

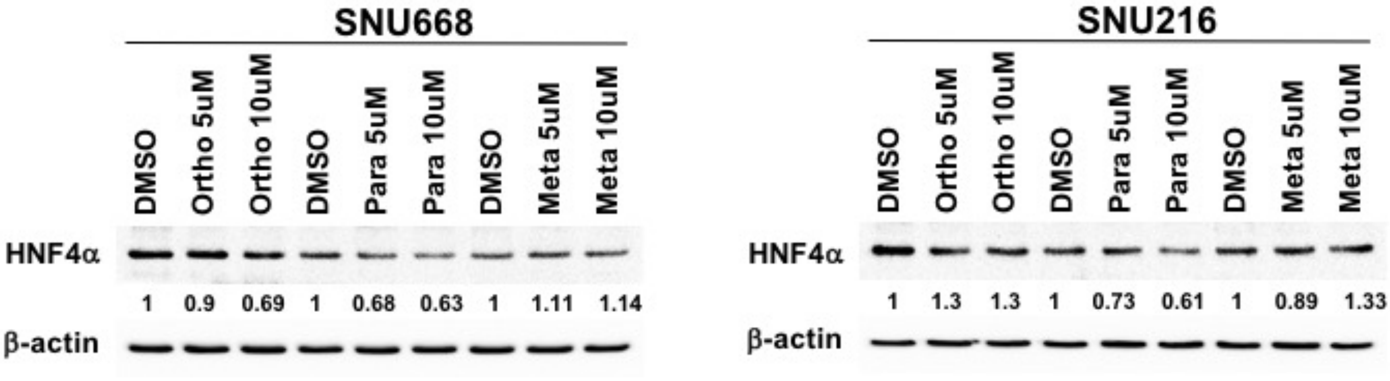

Figure S1 Western blot analysis showing HNF4a protein levels in the 2 cell lines in two concentration 5 and 10 μM of the three derivatives of BI6015.

Supporting Information, Figure S2

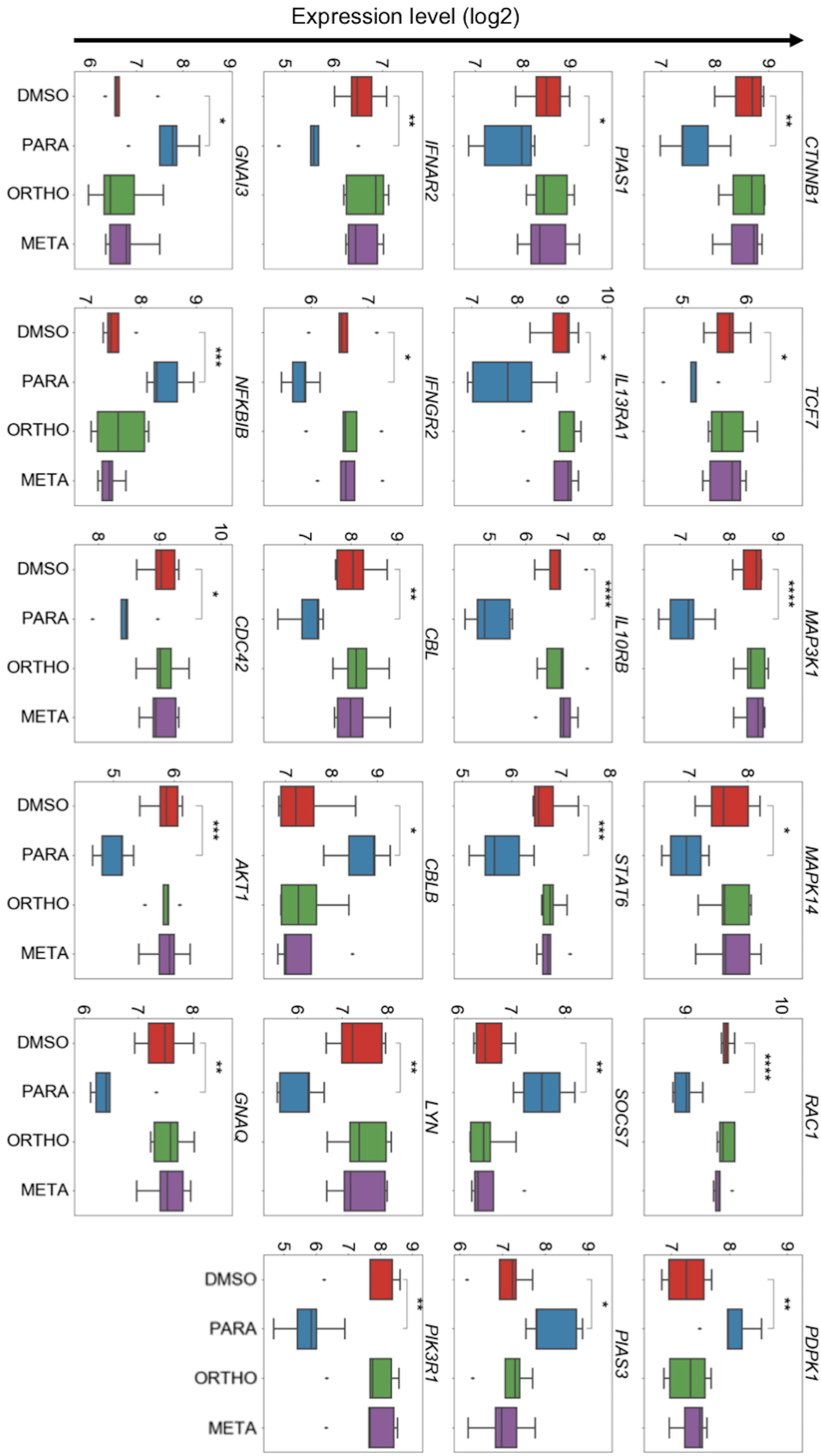

Figure S2 Commonly detected genes of para-, ortho-, and meta-groups by differentially expressed sub-pathway analysis also showed different dysregulation patterns. We tested a one-way ANOVA test with commonly detected genes by PATHOME among three treated groups, then presented twenty-three genes that were different expression patterns with significance (p-values were derived by one-way ANOVA test).
*: p-value < 0.05, **: p-value < 0.01, ***: p-value < 0.001, and ****: p-value < 0.0001.

Supporting Information, Figure S3

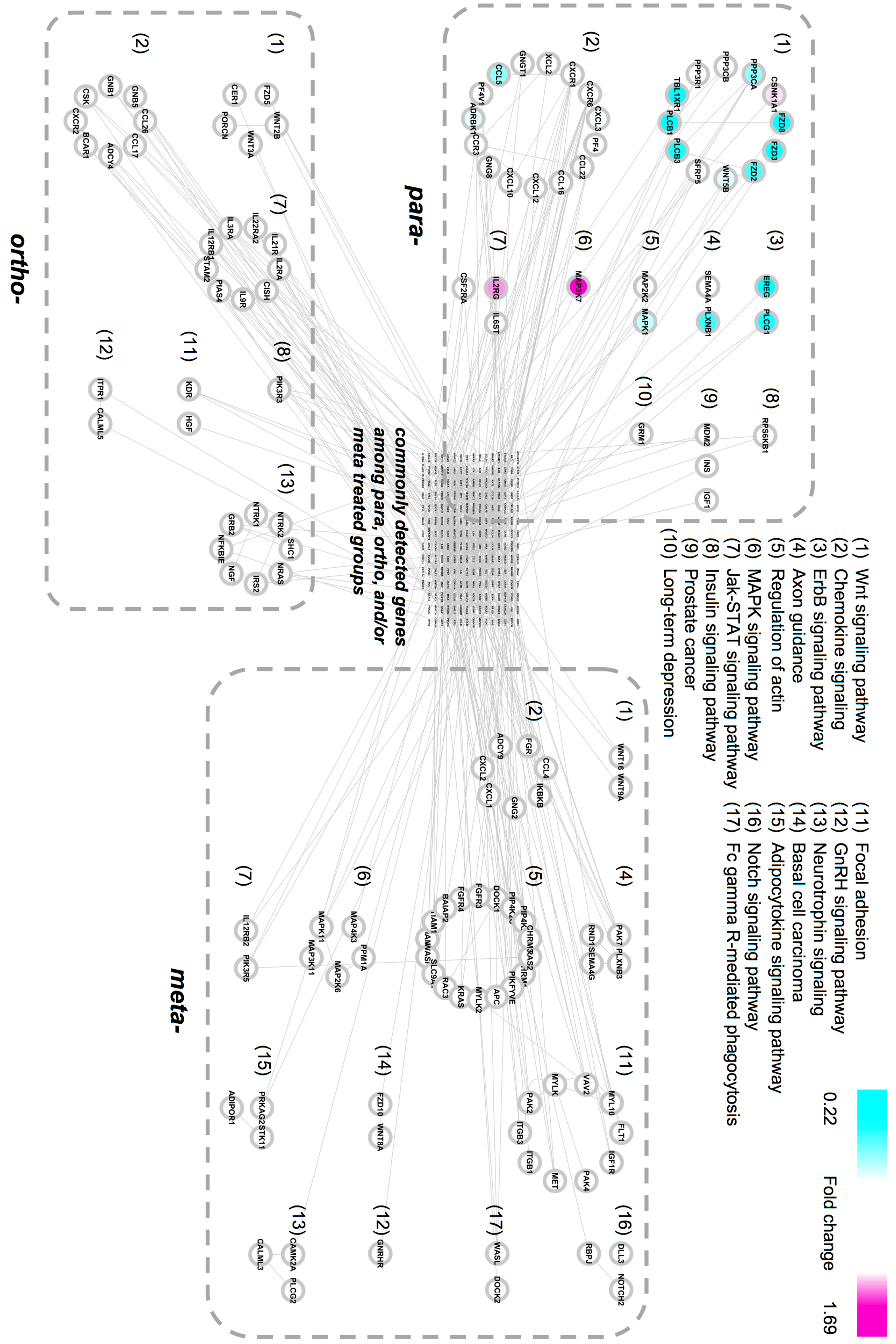

Figure S3. Differentially expressed sub-pathway analysis showed different dysregulation patterns among para-, ortho-, and meta-groups. The para treated group (top-left side) showed perturbed gene expression patterns comparing to the ortho or meta treated groups, especially in the Wnt signal pathway (marked as (1)). *FZD8*, *FZD3*, *FZD2*, *PLCB3*, *PLCB1*, *TBL1XR*, and *PPP3CA* (clock-wise) were down-regulated over DMSO groups, and CSNK1A1 was up-regulated. However, genes of ortho and meta treated groups showed small changes in the Wnt signal pathway even though those genes were identified significantly in the sub-pathway analysis. And genes in the ErbB and Axon guidance signal pathway also showed dysregulated ((3) and (4) in this figure). We performed differentially expressed sub-pathway analysis using the PATHOME (PMID: 24681952). We merged three networks from the PATHOME and mapped fold change values to genes. We arranged genes in the pathway context. Genes that were detected among three groups were condensed into small dots. (1) Wnt signaling pathway (2) Chemokine signaling pathway (3) ErbB signaling pathway (4) Axon guidance (5) Regulation of actin cytoskeleton (6) MAPK signaling pathway (7) Jak-STAT signaling pathway (8) Insulin signaling pathway (9) Prostate cancer (10) Long-term depression (11) Focal adhesion (12) GnRH signaling pathway (13) Neurotrophin signaling pathway (14) Basal cell carcinoma (15) Adipocytokine signaling pathway (16) Notch signaling pathway (17) Fc gamma R-mediated phagocytosis.

Supporting Information, Table S1

Table S1 ANOVA test result and their signal pathway context.

Detected groups[a]	Gene Symbol	KEGG signal pathway context	F value	p-value
O/P/M	CTNNB1	Wnt signaling pathway	6.935	0.0033
P/O	TCF7	Wnt signaling pathway	3.938	0.0279
P/M	MAP3K1	MAPK signaling pathway	22.739	5.16E-06
P/M	MAPK14	MAPK signaling pathway	4.281	0.0212
P/M	GNAQ	Long-term depression; GnRH signaling pathway	7.782	0.0019
O/P/M	CBL	Jak-STAT signaling pathway	6.753	0.0037
O/P/M	CBLB	Jak-STAT signaling pathway	4.913	0.0131
O/P/M	PIAS3	Jak-STAT signaling pathway	4.357	0.02
O/P/M	SOCS7	Jak-STAT signaling pathway	8.388	0.0014
O/P/M	STAT6	Jak-STAT signaling pathway	9.247	0.0008
P/M	IFNAR2	Jak-STAT signaling pathway	4.849	0.0138
P/M	IFNGR2	Jak-STAT signaling pathway	5.847	0.0067
P/M	IL10RB	Jak-STAT signaling pathway	17.531	2.60E-05
P/O	IL13RA1	Jak-STAT signaling pathway	5.231	0.0104
P/O	PIAS1	Jak-STAT signaling pathway	3.243	0.0498
P/O	PDPK1	Insulin signaling pathway; Endometrial cancer; Prostate cancer; Non-small cell lung cancer	5.588	0.0081
O/P/M	AKT1	Chemokine signaling pathway; Jak-STAT signaling pathway; Prostate cancer	12.008	0.0002
O/P/M	CDC42	Chemokine signaling pathway	4.535	0.0174
O/P/M	GNAI3	Chemokine signaling pathway	4.147	0.0236
O/P/M	PIK3R1	Chemokine signaling pathway	6.243	0.0051
P/M	LYN	Chemokine signaling pathway	7.243	0.0027
P/M	NFKBIB	Chemokine signaling pathway	9.86	0.0006
P/M	RAC1	Axon guidance	26.844	1.75E-06

[a]O: Ortho-treated group, P:Para-treated group, and M:Meta-treated group

Supporting Information, Table S2

Table S2 Differentially expressed genes of WNT/β catenin signal pathway

Gene symbol	FC PARA	p-value Para	FC Ortho	p-value Ortho	FC Meta	p-value Meta
**AXIN1**	0.603	0.001	1.069	0.415	1.033	0.633
**CTNNB1**	0.507	0.008	1.011	0.950	0.972	0.870
**DKK1**	0.093	0.001	1.049	0.923	1.076	0.889
**HDAC2**	0.596	0.001	1.061	0.571	1.071	0.456
**KAT2A**	0.300	0.005	0.946	0.834	0.971	0.913
**SKP2**	0.190	0.001	1.041	0.886	1.055	0.853
**TCF7**	0.690	0.020	1.016	0.910	0.993	0.958
**PPARD**	1.892	0.003	1.043	0.806	0.972	0.868
**CCND2**	1.517	0.032	1.074	0.167	1.105	0.079
**HDAC5**	2.167	0.015	0.970	0.901	1.000	1.000
